# Genome-wide analysis of the interaction between the endosymbiotic bacterium *Wolbachia *and its *Drosophila *host

**DOI:** 10.1186/1471-2164-9-1

**Published:** 2008-01-02

**Authors:** Zhiyong Xi, Laurent Gavotte, Yan Xie, Stephen L Dobson

**Affiliations:** 1 Department of Entomology; University of Kentucky; Lexington, KY 40546, USA; 2 Department of Entomology, Michigan State University; East Lansing, MI 48824, USA; 3 Institut des Sciences de l'Evolution. UMR-CNRS 5554, Bat 24, CC063. Université Montpellier II. Place Eugène Bataillon 34095 Montpellier Cedex 5, France; 4 Department of Statistics, University of Kentucky, Lexington, KY 40546, USA

## Abstract

**Background:**

Intracellular *Wolbachia *bacteria are obligate, maternally-inherited, endosymbionts found frequently in insects and other invertebrates. The success of *Wolbachia *can be attributed in part to an ability to alter host reproduction via mechanisms including cytoplasmic incompatibility (CI), parthenogenesis, feminization and male killing. Despite substantial scientific effort, the molecular mechanisms underlying the *Wolbachia*/host interaction are unknown.

**Results:**

Here, an *in vitro Wolbachia *infection was generated in the *Drosophila *S2 cell line, and transcription profiles of infected and uninfected cells were compared by microarray. Differentially-expressed patterns related to reproduction, immune response and heat stress response are observed, including multiple genes that have been previously reported to be involved in the *Wolbachia*/host interaction. Subsequent *in vivo *characterization of differentially-expressed products in gonads demonstrates that Angiotensin Converting Enzyme (*Ance*) varies between *Wolbachia *infected and uninfected flies and that the variation occurs in a sex-specific manner. Consistent with expectations for the conserved CI mechanism, the observed *Ance *expression pattern is repeatable in different *Drosophila *species and with different *Wolbachia *types. To examine *Ance *involvement in the CI phenotype, compatible and incompatible crosses of *Ance *mutant flies were conducted. Significant differences are observed in the egg hatch rate resulting from incompatible crosses, providing support for additional experiments examining for an interaction of *Ance *with the CI mechanism.

**Conclusion:**

*Wolbachia *infection is shown to affect the expression of multiple host genes, including *Ance*. Evidence for potential *Ance *involvement in the CI mechanism is described, including the prior report of *Ance *in spermatid differentiation, *Wolbachia*-induced sex-specific effects on *Ance *expression and an *Ance *mutation effect on CI levels. The results support the use of *Wolbachia *infected cell cultures as an appropriate model for predicting *in vivo *host/*Wolbachia *interactions.

## Background

Maternally transmitted *Wolbachia *are alpha proteobacteria that infect a wide range of invertebrates, including nematodes, mites, spiders, and an estimated >20% of insect species [1,2]. The ability of *Wolbachia *infections to spread and persist within this broad range of hosts can be attributed in part to its induction of multiple reproductive abnormalities including cytoplasmic incompatibility (CI), parthenogenesis, feminization and male killing. CI is the most commonly reported phenotype and induces developmental arrest of embryos resulting from matings between females and males that are infected with different *Wolbachia *types [3,4]. There is substantial interest in defining the molecular basis of the *Wolbachia*/host interaction, including the mechanisms of *Wolbachia *intracellular maintenance within host cells and mechanisms by which *Wolbachia *manipulate host reproduction [5,6]. In addition to basic scientific interest, applied researchers are also interested in *Wolbachia *as a potential tool for novel applied strategies including population replacement and suppression [7,8].

Similar to *Rickettsia prowazekii*, the *Wolbachia *genome contains genes encoding components of the Type IV secretion system (T4SS) [5,9], a pathogenic bacterial protein secretary pathway known to secrete various effector molecules affecting cell host physiology [10]. Presence of T4SS in *Wolbachia *genome suggests a potential alteration of host cell expression as a means of facilitating its intracellular survival and dissemination, as observed for other intracellular bacteria [11]. However, characterization of *Wolbachia *interaction *in vivo *is complicated by dynamic infection levels that are affected by host genotype and nutrition, variable tissue tropism, and *Wolbachia *expression patterns that differ with host age [12,13]. Thus, a simplified model system, such as an *in vitro Wolbachia *infection within a well characterized cell culture, could potentially provide a useful tool for studying mechanisms of the *Wolbachia*/host interaction.

*Drosophila *S2 cells are derived from embryonic phagocytic cells [14] and previously have been demonstrated to serve as a valid *in vitro *model for examining intracellular infections and as a system for gene expression studies using microarrays and RNAi technology [15-19]. In addition to characterizing cross talk between *Wolbachia *and host cells, *in vitro Wolbachia *infections are also being used for screens to identify novel drugs that impact obligate *Wolbachia *infections within medically important filarial nematodes [20-22]. The latter studies will benefit from validation of the *in vitro *system as a predictor of *in vivo *events and from an improved understanding of the *Wolbachia*/host interaction *in vitro*.

Here, we used *Wolbachia *infected S2 cells as a model system for studying the molecular mechanisms that determine the *Wolbachia*/host interaction. Initially, microarrays were used to examine for differential expression between uninfected and *Wolbachia *infected S2 cell cultures. To determine the utility of the S2 system as a predictor of *in vivo *differential expression, one differentially expressed transcript (Angiotensin converting enzyme; *Ance*) was subsequently examined in testes and ovaries of *D. simulans *and *D. melanogaster*. *Ance *acts as a peptidyldipeptidase or endopeptidase removing the C-terminal peptide from its substrate and is required for spermatogenesis in *Drosophila *[23]. Quantitative Reverse Transcriptase PCR (qRT-PCR) indicate that *Ance *is differentially expressed in infected and uninfected flies, consistent with results in the S2 *in vitro *system. Significantly higher *Ance *expression is observed in *Wolbachia*-infected ovaries relative to uninfected ovaries. In contrast, lower expression is observed in infected testes relative to uninfected testes.

*Ance *mutant flies were used to examine for potential involvement of *Ance *in the CI phenotype. The *Ance *mutant fly, designated l(2)34Eb2, was derived from EMS mutagenesis. Since both homozygotes and hemizygotes (34Eb2 and a deficiency) are male sterile [23,24], heterozygotes were employed in crosses. Comparison of crosses using *Ance *mutant flies and wild type flies revealed a significant difference in the egg hatch rate, consistent with a hypothesized interaction between *Ance *and the CI phenotype.

In addition to *Ance*, differential expression is observed with multiple transcripts related to sexual reproduction, immune response and heat stress response. The latter include genes that have been previously reported to be involved in the *Wolbachia*/host interaction. The results are discussed as a validation of the *in vitro *model and as support for examining additional genes which have been identified as variable between infected and uninfected cells.

## Results

### *Drosophila *S2 cell line infection

**Figure 1:**
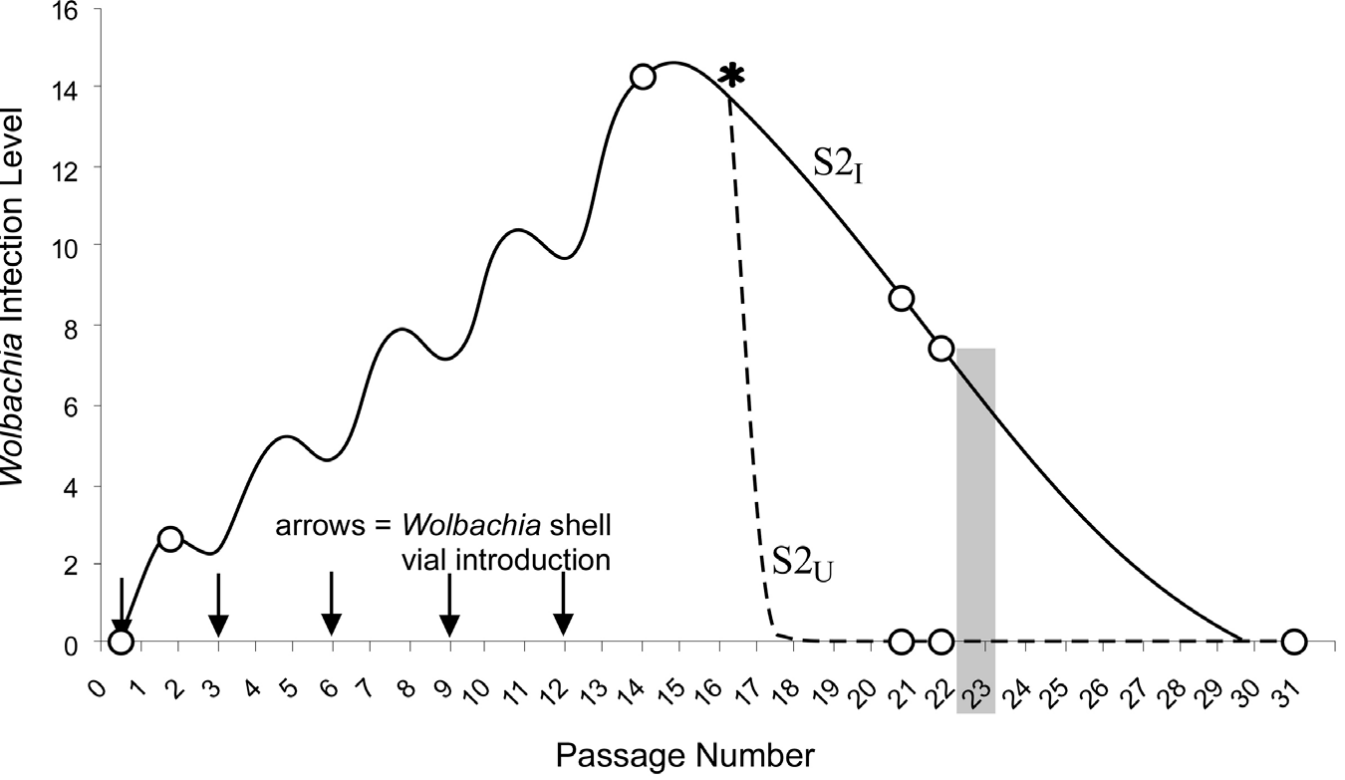
Diagram illustrating the *in vitro *infection strategy, tetracycline clearing and microarray analysis of infected (S2_I_) and uninfected (S2_U_) cell lines. The line illustrates an estimated infection level based upon periodic qPCR assays (indicated by hollow circles). The number provides a relative measure, not the absolute number of *Wolbachia*. The "waves" in the left half of the graph resulted from removal of the infection by host and enrichment of infection by shell vial introduction. Arrows indicate shell vial introduction of *Wolbachia *infection. At passage 17 (marked with asterisk), the cell line was split and one subline was tetracycline treated. At passage 23 (grey shading) the cell line was examined via microarray.

Quantitative PCR was used to measure the relative infection level in the S2_I _cell line following an initial shell vial introduction (Fig. 1). After the first *Wolbachia *introduction, the infection level in the cell line was about 10^5 ^times lower than in the DSR adult female. Therefore, the shell vial introduction was repeated sequentially as shown in Figure 1. Following five sequential *Wolbachia *introductions, the resulting *Wolbachia *infection level was increased 5.6 fold in the S2_I _cell line. To generate the uninfected S2_U _line, the S2_I _cell line was split and treated with tetracycline (Fig. 1). For transcriptomic comparison, the S2_U _cell line was used instead of the naive S2 line due to concerns that the genetic background of the S2 cell line might have been altered during the repeated transfection procedure. To avoid the potential complication caused by transcriptional differences induced by the tetracycline treatment, the microarray analysis was not conducted until six passages after antibiotic treatment. The absence of *Wolbachia *infection in the S2_U _line was confirmed by PCR with *wsp *primers (440F/691R) at passages 21 and 22 (Fig 1).

### Differential *in vitro *expression

**Figure 2:**
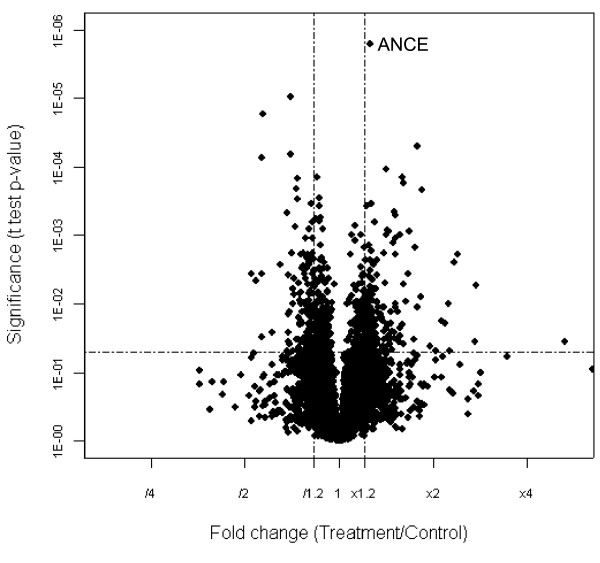
Distribution of the relative expression of 6,769 genes in the infected S2_I _versus uninfected S2_U _cell lines. Genes with differential expression are defined as those with P < 0.05 and a ± 1.2 fold change.

The Genechip contains 13,966 probe sets, of which 7,197 were found to be uninformative (signal was absent in all eight replicates). The frequency of uninformative probes is not unexpected considering the use of cells derived from embryos (Genechip includes development stage and sex-specific probe sets) and results of prior studies (use of the same platform results in 4992 probe sets with signals below the detectable threshold; [25]). Of the remaining probes, 263 sets were different between S2_I _and S2_U _cells (P < 0.05 and ≥ 1.2 fold change) (Fig. 2). A complete set of microarray data results is available at Gene Expression Omnibus [26].

To facilitate integration of the results with currently available information about the *Wolbachia*/*Drosophila *interaction, transcripts are described relative to the biological process using Gene Ontology (GO) analysis. Of the differentially transcribed probe sets, fifty are categorized as genes with unknown functions and are not discussed subsequently. The remaining 213 probe sets are identified as belonging to a particular biological process ontology, with 73 down-regulated and 140 up-regulated. After all the differentially expressed genes were enriched into GO terms, the GO terms were ranked based on Z value. The top five non-redundant Gene Ontology (GO) terms are shown in Table 1. A complete set of GO data is available in the support information (Additional File 1 and 2).

**Table 1:** The top five non-redundant Gene Ontology (GO) terms in biological process which contain the most differentially expressed genes. Up or down, up- or down-regulation after infection, respectively; T, number of genes assigned to this the GO term; M, the subset of T genes that are represented on the microarray; C, the subset of M genes observed to vary in the analysis; Z, significance score

	GO Term	T	M	C	Z
Up	Antimicrobial humoral response (sensu Protostomia)	70	20	6	7.4
	Negative regulation of cell proliferation	42	10	3	5.3
	Ion homeostasis	40	10	3	5.3
	Larvae development (sensu Insecta)	54	14	3	4.3
	Dorsal appendage formation	39	15	3	4.1

Down	Response to unfolded protein	50	15	6	12.6
	Response to chemical stimulus	248	77	6	4.7
	Tissue morphogenesis	78	16	2	3.7
	RNA splicing	101	40	3	3.2
	Positive regulation of transcription, DNA-dependent	102	34	2	2.2

### *In vivo *characterization of angiotensin converting enzyme (Ance)

**Figure 3:**
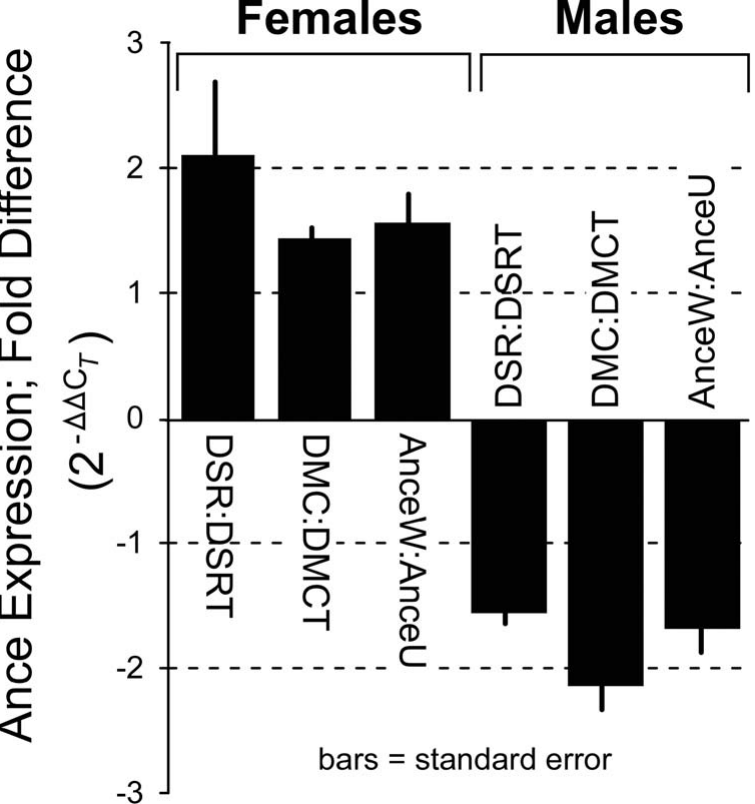
*In vivo *qRT-PCR assay of differential *Ance *expression in *Wolbachia *infected and uninfected *D. simulans *(DSR:DSRT), *D. melanogaster *wild type (DMC:DMCT) and *D. melanogaster Ance *mutant (AnceW:AnceU) flies. *Wolbachia *infection is consistently associated with higher *Ance *expression in ovaries and lower *Ance *expression in testes.

Among the probe sets on the chip, 153728_at (*Ance*) is the only gene that is differentially transcribed at the Bonferroni corrected level (Fig. 2). Therefore, *Ance *was selected for *in vivo *expression characterization. qRT-PCR was used to assay *Ance *mRNA levels in *D. simulans *and *D. melanogaster *ovaries and testis dissected from infected and uninfected flies. As shown in Figure 3, the average *Ance *expression is 2.1 fold higher in infected *D. simulans *ovaries relative to uninfected ovaries. In contrast, *Ance *expression was 1.5 fold lower in testes of *Wolbachia *infected *D. simulans *flies relative to infected testes. Similar results are observed with both the wild type (DMC) and the *Ance *mutant *D. melanogaster *flies (Fig. 3).

To examine for an interaction of *Ance *and *Wolbachia*-induced CI, crosses were conducted between wild type and *Ance*-mutant *D. melanogaster *strains that were either infected or uninfected. As shown in Table 2, no significant difference in hatch rate was observed in comparisons of the eight compatible cross types. In contrast, a comparison of the incompatible crosses revealed a higher egg hatch rate in crosses of infected wild-type males mated with uninfected *Ance *mutant females (40% hatch) relative to other incompatible crosses (26–30% hatch).

**Table 2:** CI Cross Assay Results

Expected CI Type	Cross *	Percent Egg Hatch ^†^	Egg Counted	ANOVA**
Compatible	DMCT × DMCT	68.6 ± 5.3%; 13	197	a
	DMC × DMC	66.4 ± 2.1%; 38	595	
	AnceU × AnceU	73.8 ± 3.1%; 10	248	
	AnceW × AnceW	76.1 ± 3.4%; 10	184	
	AnceW × DMC	71.7 ± 1.9%; 61	1173	
	DMC × DMCT	66.7 ± 4.3%; 15	168	
	DMC × AnceW	66.9 ± 4.0%; 9	118	
	DMC × AnceU	61.5 ± 1.5%; 10	127	

Incompatible	DMCT × DMC	28.4 ± 1.8%; 39	440	b
	DMCT × AnceW	30.4 ± 2.1%; 40	529	b
	AnceU × DMC	40.1 ± 3.4%; 46	928	c
	AnceU × AnceW	26.4 ± 1.4%; 30	795	b

*Female × Male

^† ^Average ± Standard Error; Number of Crosses

** Hatch rate was subjected to arcsine transformation before performing ANOVA, P < 0.05

### Additional differentially-expressed transcripts

**Figure 4:**
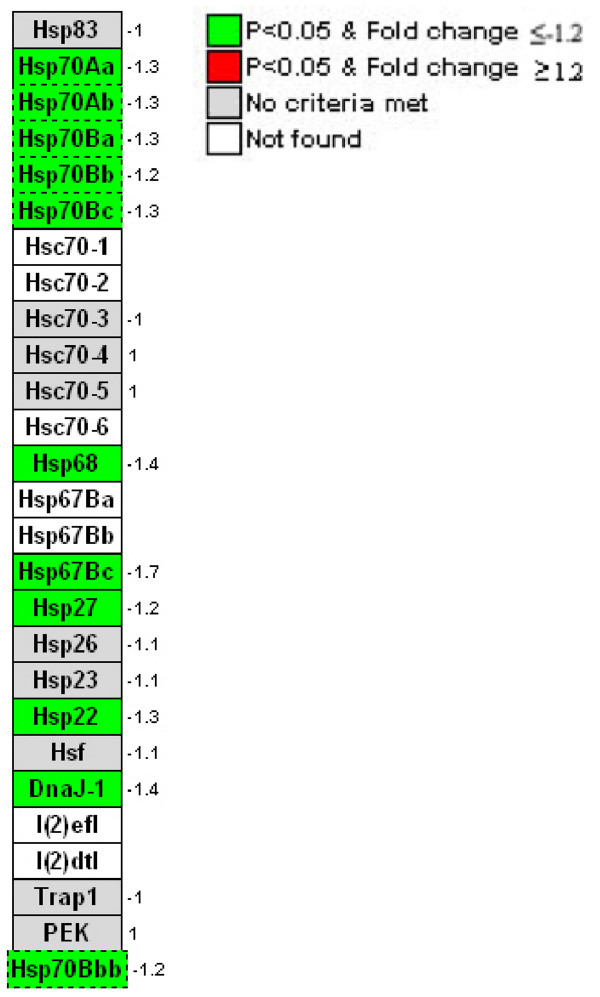
Regulation pattern of heat shock protein (*Hsp*) in *Wolbachia *infected S2 cells. All heat shock proteins in *Drosophila *are shown. Down-regulation (green) occurs in 41% (11 of 27) of known Hsps. The fold change is shown for each gene.

**Figure 5:**
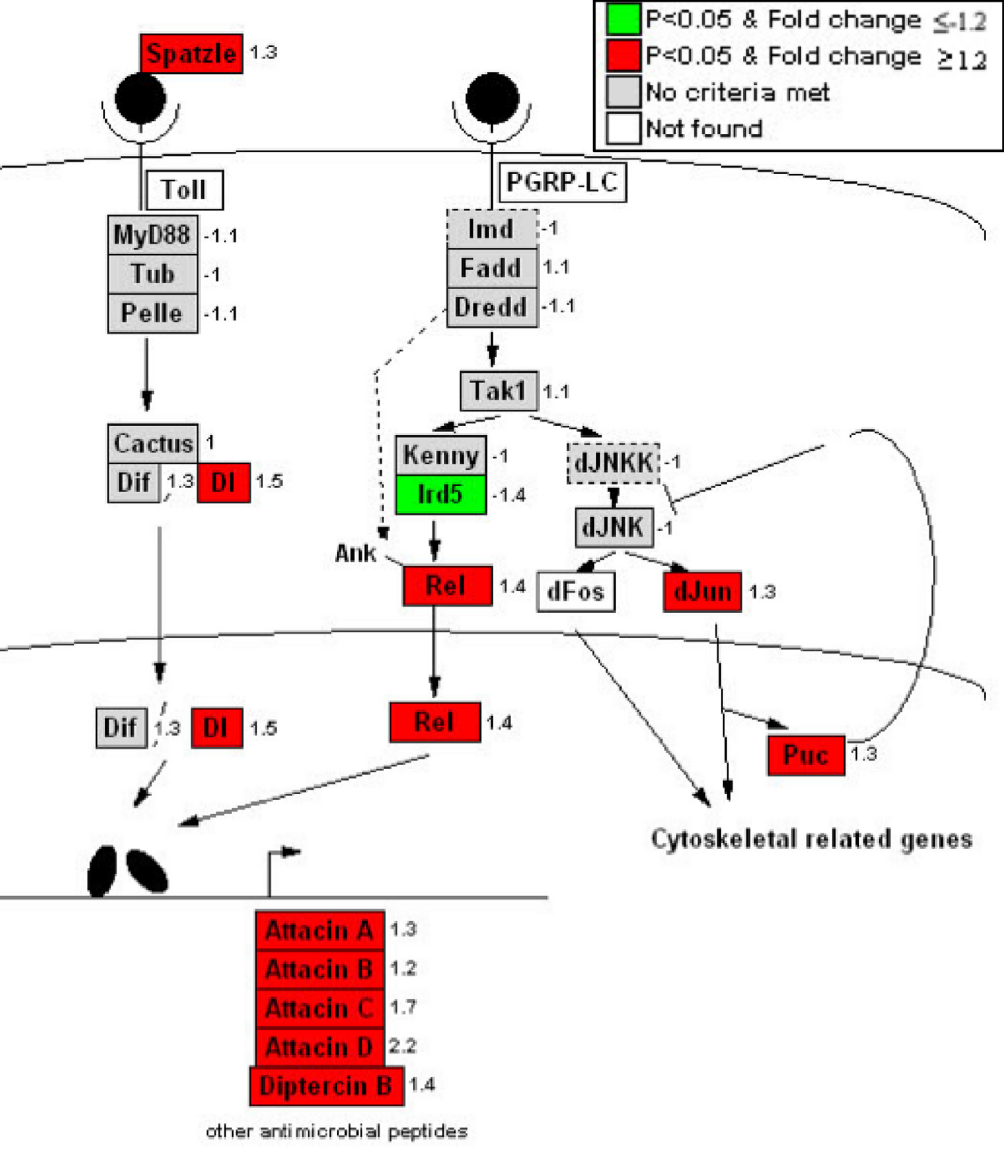
Induction of immune response to *Wolbachia *infection in S2 cells. Activation of both Toll and Imd pathways result in up-regulation of Dorsal and Relish, which might lead to a specific antimicrobial expression profile against *Wolbachia*. Negative cross talk between Imd and JNK pathway is also observed [48].

In addition to *Ance*, microarray analysis revealed nineteen differentially transcribed probe sets representing genes previously reported to be associated with reproduction (Table 3). Among these, the majority function in the different process of oogenesis, including ovarian follicle cell development, pole plasm assembly and nurse cell to oocyte cell transport. *l(2)gl*, which has been recently hypothesized as involved in the CI mechanism [6], is also up-regulated. Enrichment and sorting of the differentially-expressed probe sets into their corresponding GO term with GeneFinder revealed "response to unfolded proteins" to be the term most frequently down-regulated (Table 1). From a total of 27 heat shock proteins in *Drosophila*, eleven (41%) were lower in the *Wolbachia *infected cell culture. Up-regulation was not observed in this category (Fig. 4). "Antimicrobial humoral response" was identified as the most common up-regulated GO term following enrichment and sorting (Table 1). As shown in Figure 5, most of the genes in this GO term play a role in the immune signaling pathway, including *Toll *and *Imd*. Among these, two important regulatory proteins: Relish (*Rel*) and Dorsal (*Dl*), belong to the *NF-κB *family, and higher expression was observed in *Wolbachia *infected S2 cells. Five downstream antimicrobial peptides were also up-regulated, including *attacin *(A, B, C and D) and *diptericin *B (Fig. 5). In contrast, *ird5 *and *peroxiredoxin 2540 *were down-regulated.

**Table 3:** Classification of differentially expressed genes related to sexual reproduction based on the Gene Ontology (GO) term.

		Relative Expression Level*				
		
	GO term	Up				Down
Oogenesis	Ovarian follicle cell development (sensu Insecta)	spir	mip130	puc	ttk	brn
		Jra	loco	argos		sty
	Pole plasm assembly (sensu Insecta)	osk	spir	enc		
	Nurse cell to oocyte cell transport (sensu Insecta)	loco	spin			jagn
	Oocyte dorsal/ventral axis determination	spz	enc			
	others	gig				sxl

Other	Fertilization					noi
	Germ cell migration	wun	mod(mdg4)			
	Male gamete generation	*Ance*				

* Up or down regulated indicates higher or low expression in *Wolbachia *infected cells, respectively.

## Discussion

A genome-wide analysis of the *Wolbachia*/host interaction has been conducted *in vitro *using the *Drosophila *S2 cell line. As a simplified model system, the results are likely to under-represent *in vivo *events (Type II error), for reasons including a relatively low *Wolbachia *infection level and the reduced complexity of the embryonic cell environment in the S2 cell line. However, the use of a simplified system is not an inappropriate approach given the dearth of information currently available for the *Wolbachia*-host interaction. An important motivation for subsequent *in vivo *transcriptional assays was to address the concern that the microarray results may represent an experimental artifact. Specifically, *in vivo *characterization of *Ance *expression via qRT-PCR was used to examine the validity of the *in vitro *system as a predictor of *Ance in vivo *expression. The selection of *Ance *was based primarily upon the observed level of differential expression and prior studies describing the potential involvement of *Ance *in spermatid differentiation [23], suggesting a possible role for *Wolbachia*/*Ance *interaction. Specifically, *Wolbachia *has been observed to be associated with differentiating sperm [27], providing a potential role of sperm modification in CI [28,29].

The location of *Ance *mutation has been mapped to 34E3–5 on the second chromosome [24]. The recessive lethality associate with the mutation due to the loss of function could be rescued by expression of *Ance *in the P-transformation assay and genetic complementation test [24]. Homozygote *Ance *males are sterile. Their testes lack individualized sperm and have very few actin-based individualization complexes [23,50]. Therefore, premised upon the described involvement of *Ance *in spermatid differentiation, additional experiments were conducted to examine a model in which *Wolbachia *modifies the sperm via a pathway involving *Ance *expression. The latter experiments included characterization of *Ance *mRNA levels in testes and ovaries and examining egg hatch resulting from CI crosses of *Ance *flies.

The qRT-PCR assays demonstrate higher *Ance *expression in infected ovaries relative to uninfected ovaries. The latter observation is consistent with the higher *Ance *expression that is observed in the *Wolbachia *infected S2 cell line, which was derived from embryos. Future experiments should address whether the observed differential *Ance *expression originates within oocytes, maternally-derived ovary tissues, or both. *Wolbachia *infection was also associated with differential *Ance *expression in testes, but the variation was the reverse of that observed in ovaries. Lower *Ance *expression was observed in *Wolbachia*-infected testes relative to testes from uninfected flies. A similar pattern is observed in *D. simulans *and *D. melanogaster*. It is useful to note that the results with uninfected *D. melanogaster *are similar to prior reports in which higher *Ance *expression is observed in testes relative to ovaries (>1.6 fold increase in testes; [31,32]). However, the *Wolbachia *infection status in the prior reports is not known.

In crossing experiments, no significant differences are observed in comparisons of the compatible crosses (Table 2). However, comparison of the hatch rate resulting from the four incompatible cross types revealed a significantly higher egg hatch rate in crosses of uninfected *Ance *females and infected wild type males relative to the other incompatible crosses. It is important to note that only heterozygous *Ance *flies could be used in this experiment. Therefore, only dominant *Ance *effects would be observed. Furthermore, the possibility cannot be excluded that additional other sub-lethal mutations are co-segregating with the *Ance *mutation.

A model to describe the observed cross results would require an interaction of *Ance *with both the rescue and modification mechanisms [33]. Specifically, the *Ance*/CI model requires that: the *Ance *mutation in uninfected females results in partially offsetting the *Wolbachia*-induced modification to sperm, such that a higher frequency of modified sperm are able to escape developmental arrest, resulting in a greater number of viable larvae; and a second *Ance*/CI interaction occurs in males, which is the origin for the sperm modification. The *Ance*/CI interaction in males negates the *Ance*/CI interaction in females, such that the partial offset of CI is not apparent in crosses between uninfected *Ance *females and infected *Ance *males. Since the CI modification and rescue mechanisms and the *in vivo Ance *substrate are currently unknown [33], this precludes an ability to propose a specific mechanism to explain the above CI/*Ance *interaction model. However, observations of *Ance *expression are not inconsistent with the model proposed above. Specifically, *Ance *expression differs between *Wolbachia*-infected and uninfected *Drosophila *and that the observed variation in expression occurs in opposite directions in female or male flies. The results presented here support additional experiments to test the *Ance*/CI interaction model.

We note that a broad range of egg hatch rates have been previously reported in *Drosophila *crosses examining CI, and the egg hatch reported here is at the lower end of the reported range. Egg hatch can be affected by the experimental conditions under which females are held, and the hatch rates observed here are similar to a prior study in which females were made to oviposit in isolation [34].

In addition to *Ance*, the microarray assay identifies multiple genes that vary in expression level between infected and uninfected cells. In the following text, these are grouped based upon their involvement in oogenesis/sex determination, heat shock proteins, and genes involved in immunity.

*Wolbachia *has been reported to affect host oogenesis [35]. Multiple differentially-expressed genes were observed that have been previously shown to be involved in different processes of oogenesis, a majority of which are related to ovarian follicle cell development (Table 3). The latter is not unexpected due to the critical role of oogenesis in the maternal inheritance of *Wolbachia *and the somatic stem cell tropism of *Wolbachia *in *Drosophila *[36]. Over-expression of *l(2)gl *and *zipper *have been previously shown to mimic CI [6], leading a hypothesis that *l(2)gl *and *zipper *play a role in the CI mechanism. Consistent with this hypothesis, the results presented here show an up-regulation of *l(2)gl *in the infected cell.

Additional variation was observed in sex lethal (*Sxl*), which acts in *Drosophila *sex determination. Previously, *Wolbachia *has been shown to suppress sterility in *D. melanogaster *mutant females with protein-coding lesions in *Sxl *[37]. The results of the prior studies suggested that "*Wolbachia *does not bypass or reduce the requirement for *Sxl *in the germline in a general way, nor increase overall germline *Sxl *expression" [37]. Here, microarray data suggests that *Wolbachia *down-regulates *Sxl *expression. An additional component in the sex determination/sexual behavior cascade is transformer 2 (*Tra-2*) [38]. *Tra-2 *is suppressed (p < 0.05) in the infected cell line. However, *Tra-2 *was not included in Tables 1 or 3 since the observed 1.15 fold reduction does not satisfy designated criteria of a ± 1.2 fold change.

Multiple transcripts encoding proteins involved in heat-shock were observed to be down regulated in *Wolbachia*-infected S2 cells. A common function of heat shock proteins (*Hsp*) is as molecular chaperones that act to reduce inappropriate inter-protein interaction [39]. Prior studies have shown an ability of heat-shock to abate CI, and distinct *Hsp *isoforms have been reported in comparisons of infected and uninfected *Drosophila *[40]. Consistent with the prior study, our results indicate a down-regulation of *Hsp *in *Wolbachia*-infected cells. Thus, heat-shock may act to negate the impact of reduced *Hsp *expression in *Wolbachia*-infected flies.

*Wolbachia *infection in S2 cells is associated with the induction of antibacterial peptides. Prior studies observed that *Wolbachia *did not alter the expression of *defensin*, *diptericin *and *cecropin *in *D. simulans *or *A. albopictus *[41]. Consistent with the prior results, the microarray assays reported here did not detect changes in the expression of these three antimicrobial peptides. In contrast, *Wolbachia*-infected cells show higher expression with multiple genes involved in the *Toll *and *Imd *immune signaling pathways (Fig. 5). Relish (*Rel*) and Dorsal (*Dl*) are regulatory proteins in *Drosophila *that belong to the *NF-κB *family of transcription factors and play an important role in innate immunity [42]. *Drosophila Rel *is critical in *Imd *signaling pathways and is transcriptionally up-regulated in response to gram negative bacteria challenge [43]. An activation of the *Toll *and *Imd *immune pathways by gram-negative *Wolbachia *bacteria could be related to structurally characterized peptidoglycans in *Wolbachia *[44] that bind to the extracellular peptidoglycan recognition protein (PRGP) and stimulate the *Drosophila *innate immune signaling pathway [45].

An inhibition of additional expression products involved in immunity is also observed in the microarray assay. The expression of *ird5*, the *Drosophila *homolog of *IKK*, is down-regulated. Prior studies show that the activation of *Relish *requires *ird5 *[46] and that *Escherichia coli *survive 100 times better in *ird5 *mutant lines relative to wild-type *Drosophila *[47]. Components in the JNK pathway, *dJun *and *Puc*, were also observed to be higher in infected cells. Prior studies show negative cross talk between the JNK and *Imd *signaling pathways [48]. *Peroxiredoxin 2540 *expression was also lower in infected cells. Inhibition of superoxide production has been demonstrated to be important to survival of *Anaplasma phagocytophila*, which is another intracellular bacterium and close relative to *Wolbachia *[11]. Together, the latter suggest an ability of *Wolbachia *to evade the host immune responses.

Eighteen passages after the final shell vial transfection, *Wolbachia *infection in S2_I _could not be detected by PCR (Fig. 1). The disappearance of *Wolbachia *from the cell is consistent with microarray data showing activation of the host immune response. In previous studies, when *Wolbachia *was transferred by microinjection from *D. simulans *to *D. melanogaster*, the latter showed lower densities of *Wolbachia *than the naturally infected *D. simulans *and did not express high levels of CI [49]. Future experiments should examine the role of immune response in the establishment and maintenance of *Wolbachia *infection. For example, RNAi technology may be used to interrupt specific immune pathways in S2 cells [19]. The latter cells may then be examined for an ability to sustain *Wolbachia *infection.

## Conclusion

In summary, the results presented here show the S2 *in vitro *system to be a useful tool for examining the *Wolbachia*/host interactions that affect host range and CI phenotype and for defining the host immune response to *Wolbachia *infection and *Wolbachia *immune evasion mechanisms. Differences observed in the S2 cell culture system are consistent with prior studies examining the *Wolbachia *interaction within insect hosts. The utility of the *in vitro *system is further supported by results of the *in vivo *characterization of *Ance *expression and CI phenotype assays. The results provide support for the future examination of *Ance*, *Hsp *and immune related genes in the *Wolbachia*/host interaction. The microarray results also provide rationale for examining additional gene products that have not yet been assigned a function, but that are shown to vary between infected and uninfected cells.

## Methods

### *Drosophila *strains and Crosses

Flies used in this study include *Wolbachia-*infected and uninfected *Drosophila simulans *Riverside flies (DSR and DSRT, respectively) and *D. melanogaster *Canton (DMC and DMCT, respectively). To generate *Wolbachia *infected *Ance *flies (named 'AnceW'), male *D. melanogaster *('AnceU'; *Wolbachia *uninfected; genotype: ance [34Eb-2] Adh [D] pr[1] cn[1]/CyO, Adh [nB]; Bloomington Stock Center #3584) were mated with a *Wolbachia*-infected *D. melanogaster *strain with the CyO balancer chromosome (genotype: wg [Sp-1]/CyO; ry [506] Sb[1] P{ry [+t7.2] = Delta2–3}99B/TM6; Bloomington Stock Center #2535). Due to maternal inheritance, progeny from the above cross are *Wolbachia *infected. Subsequently, the infected *Ance *mutant line is maintained over the CyO balancer background by selecting for flies with cinnabar eye color and curly wing mutations. All flies were maintained at 25°C using standard *Drosophila *rearing conditions [50].

For the CI assay, one-day-old males were mated with three-day-old females for 12 hours in an apple juice plate container. Females were then isolated individually on a yeast coated apple juice plate to collect eggs. CI was determined as the proportion of hatching eggs, as previous reported [51]. Hatch rate was subjected to arcsine transformation before performing ANOVA.

### Establishment of *Wolbachia *infected (S2_I_) and uninfected (S2_U_) cell lines

*Drosophila *S2 cells were grown in Schneider's *Drosophila *medium supplemented with 10% heat-inactivated fetal bovine serum (Invitrogen). Cells were maintained as described previously [52,53].*Wolbachia *infection in the S2 cell line was established using a previously described shell vial technique [53]. In brief, DSR eggs were collected by standard procedures [54], homogenized and introduced into S2 cells. To increase the infection level, the shell vial technique was repeated five times sequentially at an interval of every third passage (Fig. 1). The resulting *Wolbachia*-infected S2 cell line is subsequently referred to as S2_I_. The uninfected S2_U _cell line was generated by dividing S2_I _at passage 17 into two sub-lines and tetracycline-treating one sub-line (10 μg/ml final concentration) [53] as diagrammed in Figure 1.

### Microarray analysis

The S2_I _and S2_U _cell lines were each divided at passage 23 (Fig. 1) into four independent cultures. Each culture was allowed to grow to confluency and then total RNA was extracted from 5 × 10^6 ^*Drosophila *cells from each of the four replicate preparations using the RNeasy Mini Kit (Qiagen), following the manufacturers instructions. Eight Affymetrix *Drosophila *genome chips (1.0) were used, with four chips for each of the infected and uninfected treatments. Synthesis of cRNA, labeling, hybridization, staining, washing and detection were performed according to the GeneChip Expression Analysis Technical Manual (Affymetrix). Image data was quantified using the genechip analysis microarray suite 5.0 (MAS 5.0; Affymetrix).

If all eight replicates for a particular probe set were assigned an "absent" value, the probe set was removed from further consideration. The transcript level of the remaining 6,769 probe sets were normalized (divided by the corresponding chip median) and log transformed [55]. ANOVA was performed for each probe set in SAS (SAS Institute, Cary, NC) [24]. A Bonferroni significance level was used as an initial criterion for rejecting the null hypothesis of a significant treatment effect (0.05/6769). A second arbitrary nominal threshold of α < 0.05 was used because the Bonferroni correction is overly conservative as tests are correlated [56,57]. A FDR calculation for each p was performed with QVALUE (V1.0) [58]. This threshold (P = 0.05) corresponded to a false discovery rate (FDR) of 0.46. The change was calculated as the average of four replicates. The following criteria were used to define the differential expression caused by treatment: P value < 0.05 and ≥ 1.2 fold change.

### Gene ontology analysis

MAPPFinder [59] was used to enrich, rank and classify the differentially expressed genes based on Gene Ontology (GO) [60] information of each gene. To visualize the differential expression pattern, before Z value ranking, all gene ontology terms were further filtered manually with the criteria: the number of changed genes: >2; the number of measured genes: >10; the percent of changed genes: >5%; the percent of gene presented on the chip: >20%. If the annotation for interested genes were missed in the gene database of GenMapp, they were examined in Affymetrix NetAffya analysis center [61]. The map for heat shock protein (*Hsp*) was built by MAPPBuilder [59] based on the information from GO and the Flybase[62]. The map for *Drosophila *immune response pathway was built by GeneMAPP based on the previous reports [63,64].

### Quantitative PCR and quantitative RT-PCR

Quantitative PCR (qPCR) was performed to characterize the relative *Wolbachia *infection level in the S2 cell lines and flies. The protocol was similar to prior qPCR amplification using the single-copy *wsp *and *su(fk)C *genes of bacterial and host origin, respectively [65]. S2 cells were quantified using a hemocytometer to obtain 10^6 ^cells. The S2 cells or DSR females were homogenized in 100 μl STE with 0.4 mg/ml proteinase K to extract DNA as previously described [66].

For qRT-PCR, RNA extractions were performed on groups of 10 ovaries or 10 testes dissected from one-day post eclosion infected and uninfected *Drosophila *adults using the RNeasy Mini Kit (Qiagen). DNA contamination was removed with RNase-Free DNase Set (Qiagen). RNA quality and quantity was checked with NanoDrop ND-100 spectrophotometer (NanoDrop Technologies, Inc.). Synthesis of cDNA was performed with Superscript II Reverse Transcriptase (Invitrogen) using specific primer for *Ance *(AnceQ F 5'-CGGTCACGTTCGATTGGTTG-3' and AnceQ R 5'-CTTCGGTTTCCACGTTGGTTC-3') and *Actin *gene (ActinQ F 5'-GCTGACCGTATGCAAAAGG-3' and ActinQ R 5'-GCTTGGAGATCCACATCTG-3'). Primers were designed based upon *D. simulans *genbank sequences for *Ance *and *Actin *(genbank accession number: NM_057696 and NM_079486, respectively]. qRT-PCR was performed separately with the AnceQ F/R and ActinQ F/R primer pairs using a Miniopticon system (BioRad) with a Platinum SYBR Green qPCR superMix (Invitrogen). qRT-PCR reactions were conducted using a 2 minute step at 50°C, 2 minute step at 95°C and 40 cycles of 15 seconds at 95°C and 30 seconds at 56°C. A fluorescence measurement was made at the end of each cycle. A melting curve analysis was performed at the end of the amplification program to examine for primer-dimers or nonspecific amplification. Assays were performed on two (*D. simulans *and *D. melanogaster *wild type) or three (*D. melanogaster Ance *mutants) independent experiment replicates for each sex and infection type. As an examination for variability, duplicate qRT-PCR reactions were performed for each set of ovaries or testes with both the *Ance *and *Actin *primers. Relative expression of *Ance *gene was calibrated against Actin using the ΔΔC_T _calculation method [67] with:

ΔΔC_T _= (C_T,Ance _- C_T,Actin_)_infected _- (C_T,Ance _- C_T,Actin_)_uninfected_

$$\text{Expression variation}=2^{-\Delta \Delta C_{T}}$$

For comparisons of males and females, the above was modified as follows:

ΔΔC_T _= (C_T,Ance _- C_T,Actin_)_male _- (C_T,Ance _- C_T,Actin_)_female_

## List of Abbreviations used

*Ance*, Angiotensin Converting Enzyme; CI, Cytoplasmic Incompatibility; DMC, *Drosophila melanogaster *Canton; DMCT, *Drosophila melanogaster *Canton Treated; DSR, *Drosophila simulans *Riverside; DSRT, *Drosophila simulans *Riverside Treated; GO, Gene Ontology; qRT-PCR, Quantitative Reverse Transcriptase PCR; T4SS, Type IV Secretion System.

## Authors' contributions

ZX established the *Wolbachia *infection in *Drosophila *S2 cell line, prepared the RNA for micorarray assay, performed the gene ontology analysis and drafted the manuscript. LG designed *Ance *primers, measured the *Ance *expression by q-RT PCR, and conducted crosses and the related data analysis. YX performed the microarray and CI data analysis. SD conceived of the study, participated in its design and coordination and helped to draft the manuscript. All authors read and approved the final manuscript.

## Supplementary Material

Additional file 1 All the Enriched GO term generated from up-regulated genes induced by *Wolbachia *infection. Click here for file

Additional file 2 All the Enriched GO term generated from down-regulated genes induced by *Wolbachia *infection. Click here for file
